# The effect of high blood pressure-health literacy, self-management behavior, self-efficacy and social support on the health-related quality of life of Kazakh hypertension patients in a low-income rural area of China: a structural equation model

**DOI:** 10.1186/s12889-021-11129-5

**Published:** 2021-06-10

**Authors:** Qinghua Zhang, Feifei Huang, Lei Zhang, Shasha Li, Jingping Zhang

**Affiliations:** 1 School of Medicine, Hu Zhou University, 759-Second Ring East Road, Huzhou, 313000 Zhejiang China; 2 School of Nursing, Fu Jian Medical University, Fu Zhou, Fujian China; 3 First Affiliated Hospital of Xinjiang Medical University, Urumqi, Xinjiang China; 4 Nursing Psychology Research Center of Xiangya Nursing School, Central South University, 172 Tong Zi Po Road, Changsha, 410000 Hunan China

**Keywords:** Kazakh, Hypertension, Health literacy, Health-related quality of life, Structural equation model

## Abstract

**Background:**

Health-Related Quality of Life (HRQoL) of hypertensive patients is not only affected by the disease itself but also by some subjective factors. Low health literacy is prevalent among ethnic minorities**.** Considering the Kazakh-Chinese people have the highest prevalence of hypertension in Xinjiang, and the High Blood Pressure-Health Literacy (HBP-HL) has not been included in the study of HRQoL. The synergistic effects and the potential mechanism HBP-HL, self-management behavior, therapeutic adherence, self-efficacy, social support on HRQoL remain unclear. This study aimed to introduce the HBP-HL, and develop a structural equation model (SEM) to identify the factors influencing of the HRQoL among Kazakh hypertensive patients.

**Methods:**

The data was obtained by questionnaire survey and physical examination in 2015. Patients with hypertension were recruited through random cluster sampling in Kazakh settlements in Xinjiang. Firstly, the blood pressure was measured. Then the one-for-one household interviews were conducted by Kazakh investigators. The questionnaires regarding HBP-HL, HRQoL, self-management behavior, therapeutic adherence, self-efficacy, and social support were used to collect data. Finally, SEM was constructed, and *p* ≤ 0.05 was taken as significant.

**Results:**

The data was analysed by SPSS18.0 and AMOS18.0 software. 516 Kazakh hypertension patients were recruited, and 94.4% of them had a relatively low HBP-HL score. The mean standardized scores of HRQoL, self-management, therapeutic adherence were poor; they were 63.5, 66.2, and 64.4, respectively. But 96.1% and 98.3% of the participants had high levels of self-efficacy and social support. The SEM of the HRQoL had a good overall fit (χ^2^/df = 2.078, AGFI = 0.944, GFI = 0.968, CFI = 0.947, IFI = 0.949, RMSEA = 0.046). The model indicated that the HBP-HL has the highest correlation with HRQoL, following with self-management behavior, social support, and self-efficacy.

**Conclusions:**

Low HBP-HL is a major influenced factor of HRQoL among Kazakh hypertensive patients. Future programs should consider HBP-HL as the breakthrough point when designing targeting intervention strategies.

## Background

Hypertension is the most significant risk factor for cardiovascular disability and death that affect a high proportion of people worldwide. Especially in low- and middle- income countries, for example China, Brazil, India and Mexico, the economic burden of cardiovascular disease (CVD) and hypertension contributed together 50% of the total number of economic estimates identified [1]. The Chinese Cardiovascular Disease Report 2018 indicates that about 245 million patients with hypertension in China, which has become a major public health problem. In Xinjiang, China, Kazakh mainly live in farm and pastoral areas due to unique lifestyle characterized by grazing and farming. Kazakh-Chinese people have the highest prevalence of hypertension (36.9%), followed by Han (33.7%) and Uygur (26.1%) [2]. The awareness and treatment of hypertension were obviously improved, but the control rate(12.6%) remain was extremely low in Kazakh-Chinese people [3]. Hypertension has become one of the major public health concerns among Kazakh-Chinese people.

Health-related Quality of Life (HRQoL) refers to how well a person functions in their life and his or her perceived wellbeing in physical, mental, and social domains of health [4]. HRQoL has become an important outcome measure indicator in health care fields, and is commonly used as an effective assessment of any disease management plan and health status [5, 6]. Comparing with previous objective indicators such as mortality, cure rate, and morbidity, the measurement of HRQoL can comprehensively and accurately assess the health status and prevention effects of chronic diseases such as hypertension [7, 8]. It not only reflects physical health but also psychological, social, and emotional health. Furthermore, it describes the appearance of a disease and also indicates the consequences of a disease or treatment [9, 10]. Many studies have shown that the HRQoL of patients with hypertension is lower than that of patients with normal blood pressure, regardless of physical health or mental health [5, 11–14].

HRQoL of hypertensive patients is not only affected by the disease itself but also by some subjective factors such as health literacy, self-management and psychological factors.

High Blood Pressure-Health Literacy (HBP-HL) refers to the ability of hypertensive patients to acquire, understand, and deal with hypertension-related knowledge as well as the medical services needed to control diseases [15]. Previous studies have indicated HBP-HL as a powerful indicator for predicting the health status of people, which is highly correlated with morbidity, mortality, life expectancy, and HRQoL of people with hypertension [16], especially in ethnic minorities [17, 18]. Low health literacy usually leads to a series of negative health outcomes [19]. The existing literature has also demonstrated that HBP-HL is an independent predictor of blood pressure control [20, 21], and emphasized that health care providers should evaluate HBP-HL level when they meet the patients with hypertension for the first time in order to provide tailored interventions [22, 23]. However, HBP-HL has not been introduced into the study of the HRQoL of patients with hypertension, especially among Kazakh hypertension patients in rural China.

Self-management is a dynamic process in which individuals actively apply cognitive and behavioral strategies to manage their own thoughts, emotions and behaviors [24]. Effective self-management can not only encourage patients to actively monitor their condition, and regulate their behavior and emotions, but also can improve their HRQoL [25–27].

Therapeutic adherence refers to patients’ compliance with medical conventions and active adoption of health-promoting behaviors [28]. Studies have demonstrated that the therapeutic adherence is typically higher among patients with acute illness, compared to those with chronic ones [29, 30]. Poor therapeutic adherence is strongly related to the uncontrolled blood pressure among hypertensive patients [31]. Mollaoglu found that a significant positive correlation between therapeutic adherence, self-efficacy and HRQoL [32]. Hanus suggested that therapeutic adherence can’t predict HRQoL, although patients with high adherence scores had better HRQoL [33].

Self-efficacy is an important determinant of intention and behavior. On the one hand, self-efficacy can predict HRQoL, and low self-efficacy usually leads to low HRQoL [34]. On the other hand, as a mediating variable, self-efficacy can promote the improvement of self-management level, and ultimately better the HRQoL in people suffering from chronic disease [35]. Evidence suggests that patients with high self-efficacy had better blood pressure control [36].

An increasing number of studies demonstrate that social support is significantly related to self-efficacy, the interaction between the two factor can predict the HRQoL, and is one of the important mediating factors in determining the level of HL and self-management behavior [37, 38]. For hypertension, social support not only can improve the therapeutic adherence, help to control blood pressure [39], but also contribute to optimize the HRQoL in hypertensive patients [40].

**Fig. 1:**
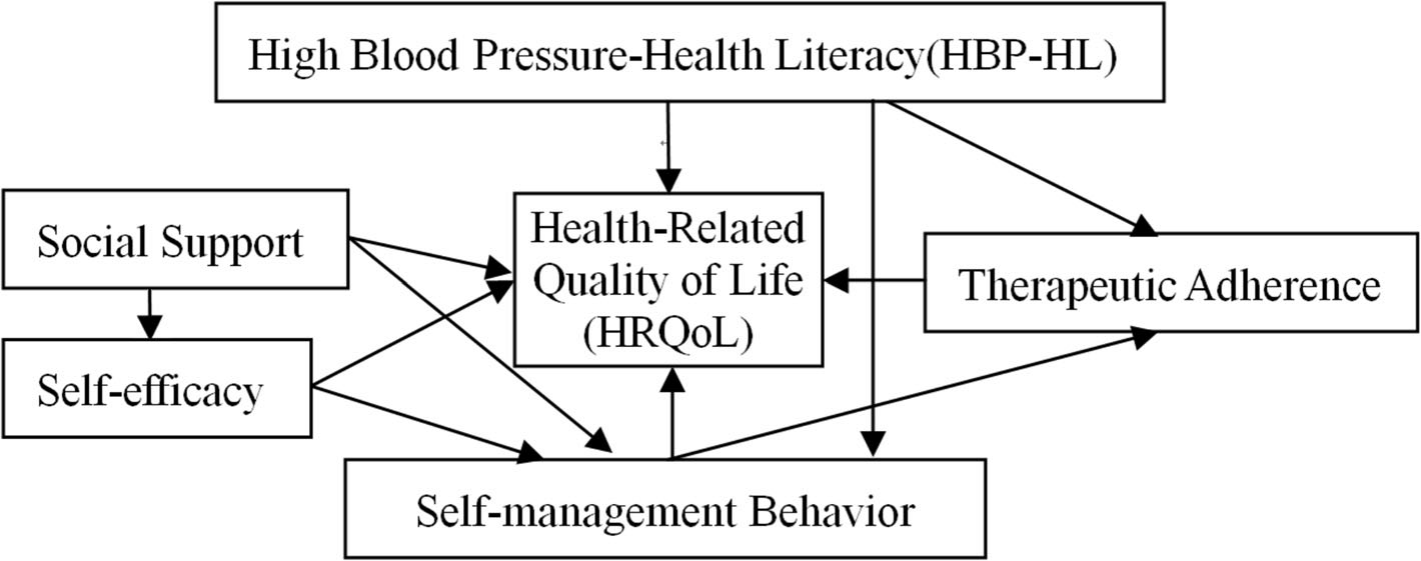
Initial Hypothesis Model of HRQoL among Kazakh hypertension patients in China. (M1)

Based on the above mentioned existing literatures, HBP-HL, self-management behavior, therapeutic adherence, self-efficacy, social support and HRQoL are related and have complex relationships. However, their synergistic effects on HRQoL and the potential mechanism remain unclear. Only one article using the structural equation model (SEM) explored the relation between health literacy and HRQoL in hypertension patients and found that enhancing the level of health literacy and self-management might improve the HRQoL of patients with hypertension, but the health literacy is universal and lack of pertinence [41]. Therefore, this study aims to introduce the conception of HBP-HL, and to expound the status quo of HRQoL, HBP-HL, self-management behavior, therapeutic adherence, self-efficacy, and social support of Kazakh hypertension patients in rural of Xinjiang. Eventually develop a SEM to explore the factors influencing the HRQoL of Kazakh hypertensive patients, and clear the direct and indirect effect of factors affecting HRQoL. Figure 1 shows the initial hypothesis model (M1) in this study.

## Methods

### Research participants

The Kazakh hypertension patients in the rural area of Xinjiang were recruited through random cluster sampling in 2015. First, based on the census data of Xinjiang, a list of the main Kazakh residence areas in Xinjiang was generated; one research site (from Urumqi counties) was randomly selected from this list. Second, five towns/town ships were randomly selected from the selected Urumqi counties. In the selected towns, only Kazakh residents who were diagnosed with hypertension at the age of 18 years or older were eligible for this study. Finally, hypertensive patients diagnosed in Kazakh settlements in Baiyanggou, Toli Ranch, Gangou Township, Xiaoquzi, and Sardanban Townships in Urumqi County, Xinjiang who met the following inclusion criteria were recruited: (1)18 years old or older; (2) Meeting the criteria for the diagnosis of essential hypertension, systolic blood pressure (SBP) ≥140 mmHg, and / or diastolic blood pressure (DBP) ≥90 mmHg [42]; (3) Blood pressure (BP) levels that have not reached the above-mentioned diagnostic criteria but have a history of hypertension, and are currently taking antihypertensive drugs; (4) Normal cognitive ability, and the capability to independently (or through the investigator) read and fill in the required research questionnaire; and (5) Agreement by Kazakh patients to participate to this study and sign the inform consent. However, if the patients met any of the following criteria, they were excluded: (1) Mental disorder and/or mental retardation; (2) Serious social dysfunction (patients with an inability to communicate); (3) Participation in other research projects in the past 1 month; (4) Profession in medical field and/or relation to medical professionals; (5) Cancer patients who received radiotherapy or chemotherapy in the past 6 months; (6) Patients with various types of secondary hypertension.

### Data collection

A general rule of thumb is that models with 5 or more items were analysed, at least 500 observations sample were needed [43]. Then adding 10% non-response rate, sample size became 550.

The one-for-one household interviews were conducted by Kazakh investigators. The questionnaires were further checked for missing data. If any missing information was identified, further information was collected via phone call to the numbers provided by the participants or one more visit to the participants’ house. However, a group of participants have missing data; they were unreachable later on.

The survey collected general information (including demographic characteristics, such as gender, age, education level, marital status, occupation, and annual family income) from the participants. HBP-HL, HRQoL, self-management behavior, therapeutic adherence, self-efficacy, and social support were also measured. All these scales were assembled into one questionnaire.

#### Instruments

##### Chinese-High Blood Pressure-Health Literacy Scale (C-HBP-HLS) [44]

consists of 15 items in 5 dimensions (Print Health Literacy, Medication Label, Understanding Ability, Newest Vital Sign Test, and Avoiding Food Allergy), which was developed and validated by Kim MT, et al. in 2012 [45]. According to the Test of Functional Health Literacy in adults (TOFHLA) scoring system [46], the final scale ranged from 0 to 60 points, and a higher score reflects higher the HBP-HL level. In addition, according to the TOFHLA classification criteria [47], the patients were further categorized into three HBP-HL levels: lack (< 32 points), medium (32 to 40 points), and sufficient (≥ 40 points). The scale-level content validity index was 0.85. Cronbach’s α of the overall scale was 0.78, and the test-retest reliability was 0.96.

##### HRQOL Instruments for Chronic Disease-Hypertension (QLICD-HY) [48]

The QLICD-HY includes 30 items, it was divided 4 dimensions (Physical Function, Psychological Function, Social Function and Specific Module), and was used to evaluate HRQoL for patients with hypertension, which was developed and validated by Wan C, et al. in 2012 [48]. The scores for each patient was calculated with the specific formula: standardized score = (Raw Score-minimum) × 100/(Max-Min). The higher the total scores reflect better HRQoL. The test–retest reliability coefficient for the overall score was 0.89, the Cronbach’s α for these four domains ranged from 0.66 to 0.88.

##### Hypertension Patients Self-Management Behavior Rating Scale (HPSMBRS) [49]

The scale includes 33 items, which was developed and validated by F Zhao, Q, et al. in 2012 [49]. The higher the total scores reflect better Self-management levels. Cronbach’s α of the overall scale was 0.914.

##### Therapeutic Adherence Scale for Hypertensive Patients (TASHP) [50]

consists of 25 items, which was developed and validated by Tang H, et al. in 2011, and was used to assess the compliance of hypertension patients in recent months. A higher scores mean better therapeutic adherence*.* Cronbach’s α of the overall scale was 0.86, and the test-retest reliability was 0.96.

##### Self-efficacy of chronic diseases scale

Self-efficacy for managing chronic disease was assessed by the Stanford 6-item Scale, which was developed and validated by Lorig KR, et al. in 2001 [51]. The scale is rated on a 10-point scale ranging from “not at all confident” to “totally confident”. It has been widely used in many countries and has good reliability and validity [52]. It was composed of 6 items in 2 dimensions (Symptom management and disease generic management), and a total scale is the sum of the average scores of each item. The higher the total scores reflect better self-efficacy. According to the total scores, a score less than 4.0 indicated low self-efficacy, of 4 to 7.9 indicated moderate self-efficacy, and more than 8.0 indicated high self-efficacy.

##### Social Support Rating Scale (SSRS) [53]

10 items with 3 dimensions and the total scores are the sum of each item, which was developed and validated by Xiao SY, et al. in 1994 [53]. It has been used widely in China and has a good reliability and validity. A higher scores reflect better social support. The social support was categorized into four groups based on the total scores, which were: low (< 20 points), medium (20 to 30 points), high (30 to 40 points), very high (≥ 40 points). The retest reliability is 0.92; the consistency of each item is 0.89–0.94 [54].

All original scale scores were standardized to make the comparable standardized scores = (factor per capita value/the full number of each item) × 100, with the exception of QLICD-HY.

#### Blood pressure measure

The AU-621 (A & D Medical Life source, Japan) electronic sphygmomanometer [Ande Electronics (Shenzhen) Co., Ltd.] was used to measure the blood pressure of the right upper limb of the patient. The AU-621 was calibrated every 6 months. Before the measurement, the participants were asked to rest for at least 5 min. The sphygmomanometer’s cuff was placed on the right upper limb elbow of the patient two consecutive times, at least 30 s apart, and averaged.

### Statistical analysis

All statistical analysis was performed using SPSS18.0 and AMOS18.0 software. The continuous variables (SBP, DBP, and the scores of HBP-HL, HRQoL, self-management behavior, therapeutic adherence, self-efficacy, and social support) were reported as mean ± standard deviation. The categorical variables (Age, gender, marriage, occupation, education and the annual family income level) were reported in percentage or composition ratio. The path analysis model was used to create the structural equation model (SEM) for predicting the HRQoL of Kazakh hypertension patients with hypertension (a = 0.05 for entry into the model, and a = 0.10 for excluding from the model). *P* ≤ 0.05 indicates statistical significance. The following indexes were used to evaluate the goodness-of-fit of hypothesized models:χ2 /df < 3, Root Mean Square Error of Approximation (RMSEA < 0.08), Goodness-of-fit Index (GFI > 0.90), Adjusted Goodness-of-fit Index (AGFI> 0.90), Incremental fit Index (IFI > 0.90), Comparative fit Index (CFI > 0.90) [55].

## Results

The study was conducted in 2015. Overall, 550 patients who met the eligibility criteria, in the selected townships from the rural area of Xinjiang. Among them, 24 invalid questionnaires (with incomplete data) were excluded; 516 questionnaires were valid, with a return rate of 93.82%.

### Demographic characteristics and BP level of the participants

Among the 516 included Kazakh hypertension patients in the rural area, 239 were male (46.3%), and 277 were female (53.7%). The mean age of the participants was 58.14 ± 12.05 years old. The majority of the participants were farmers and herdsmen (87.60%). In addition, around 70% participants only attended junior high school or below, and about two-thirds of the participants have an annual family income of less than RMB 10,000 yuan (75.6%).The average SBP was 156.26 ± 24.40 mmHg, and the average DBP was 87.55 ± 14.73 mmHg for the participants (see Table 1).

**Table 1:** Demographic characteristics of Kazakh hypertension patients

Variables	Group	N(%)
**Age (years)**	<50	130(25.19)
	50–60	138(26.75)
	60–70	148(28.68)
	≥70	100(19.38)
**Gender**	Male	239(46.32)
	Female	277(53.68)
**Education level**	Less than high school	359(69.57)
	High school or higher	157(30.43)
**Marital status**	Married	431(83.53)
	Unmarried/ Divorce/Widowed	85(16.47)
**Occupational status**	farmers and herdsmen	452(87.60)
	Others	64(12.40)
**Family annual income**	< 10,000RMB	390(75.58)
	10,000 ~ 30,000RMB	103(19.96)
	≥30,000RMB	23(4.46)
**duration of hypertension (years)**	≤5	332(64.3)
	>5	184(35.7)

### HRQOL, HBP-HL, self-management behavior, therapeutic adherence, self-efficacy, and social support

The standardized HRQoL score of Kazakh hypertension patients in rural areas was 63.5 points. The standardization scores of each dimension of QLICD-HY from high to low were: the psychological function (68.7 points), the social function (66.1 points), the hypertension specific module (61.5 points), and the physical function (57.2 points). The standardized HBP-HL score was 24.2 points. Overall, 487 patients (94.4%) lacked HBP-HL, 7 patients (1.4%) had a medium level of HBP-HL, and only 22 patients (4.2%) were considered to be sufficient in HBP-HL. The standardized scores of self-management behavior and therapeutic adherence were 66.2 points and 64.4 points, respectively. The standardized score of self-efficacy was 64.0 points, including 20 cases (3.9%) with low self-efficacy, 422 cases (81.8%) with medium self-efficacy, and 74 cases with high self-efficacy (14.3%). The standardized social support score was 76.0 points, including 1 case (0.2%) with low social support, 8 cases (1.6%) with medium social support, 172 cases (33.3%) with high social support, and 335 cases (64.9%) with very high social support (see Table 2).

**Table 2:** Current situation of HRQoL, HBP-HL, Self-management behavior, Therapeutic Adherence, Self-efficacy and Social support

Variable	Min	Max	Mean ± SD	Standardized Score	95%CI
**Score of HRQoL**	76	210	166.41 ± 22.21	63.52	164.49 ~ 168.33
Psychological function	16	55	41.23 ± 8.45	68.70	40.05 ~ 41.96
Social function	19	49	40.07 ± 4.43	66.07	39.69 ~ 40.45
Hypertension specific module	24	79	58.82 ± 9.52	61.50	58.00 ~ 59.65
Physical function	11	39	26.29 ± 4.92	57.16	25.87 ~ 26.72
**HBP-HL**	5	58	14.51 ± 8.56	24.18	13.77 ~ 15.25
**Self-management Behavior**	56	165	109.28 ± 19.75	66.23	107.57 ~ 110.99
**Therapeutic Adherence**	45	122	80.44 ± 17.32	64.35	78.94 ~ 81.93
**Self-efficacy**	1.25	10	6.40 ± 1.40	64.00	6.29 ~ 6.52
**Social Support**	19	54	41.04 ± 4.78	82.08	40.62 ~ 41.45

*HRQoL* Health-related quality of life, *HBP-HL* High Blood Pressure-Health Literacy

### Construction and testing of structural equation model of HRQoL

Correlation analysis (see Table 3) showed that HRQoL had a significantly positive correlation with HBP-HL, self-management behavior, therapeutic adherence, social support, and self-efficacy (*P* < 0.001). Based on the results mentioned above, and the literature, the initial hypothesis model (M1) of HRQoL, HBP-HL, self-management behavior, therapeutic adherence, self-efficacy, and social support of Kazakh hypertension patients in rural areas were constructed (see Fig. 1).

**Table 3:** Correlations among HBP-HL, Self-management behavior, Therapeutic Adherence, Self-efficacy, social support and HRQoL

	HRQoL	HBP-HL	Self-management Behavior	Therapeutic Adherence	Self-efficacy
**HBP-HL**	0.212**				
**Self-management Behavior**	0.243**	−0.050			
**Therapeutic Adherence**	0.146**	−0.005	0.692**		
**Self-efficacy**	0.257**	−0.006	0.185**	0.199**	
**Social Support**	0.225**	0.051	−0.008	− 0.042	0.079

*HRQoL* Health-related quality of life, *HBP-HL* High Blood Pressure-Health Literacy

**P<0.01

**Fig. 2:**
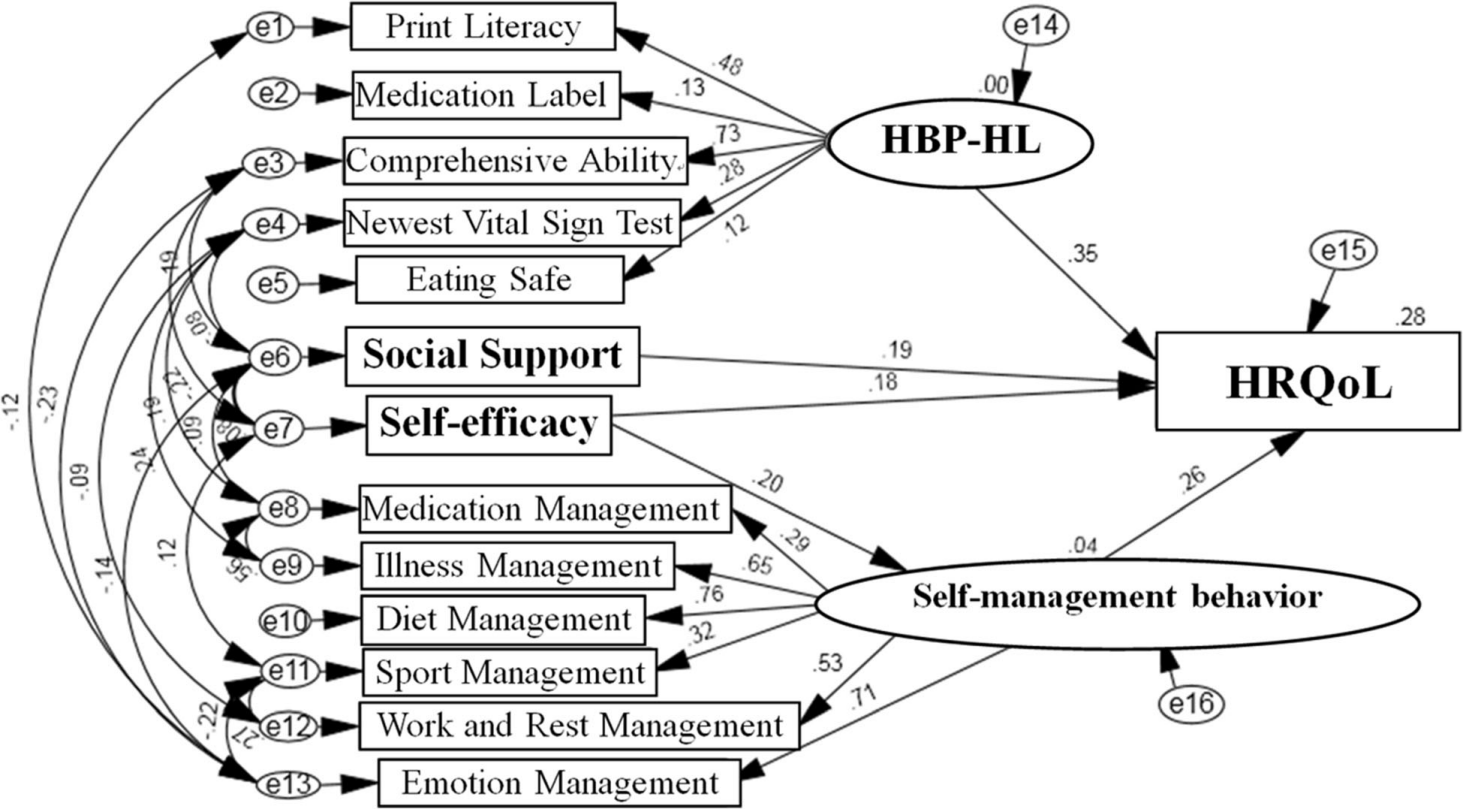
Structural Equation Model of HRQoL among Kazakh hypertension patients. (M2)

Next, the maximum likelihood method was used for parameter estimation. According to the revised index, standardized regression coefficient (path coefficient) and literature data, the paths that did not reach a significant level and were unreasonable, were deleted. Thus, the paths between social support and self-management behavior, social support and self-efficacy, HBP-HL and self-management behavior, self-management behavior and therapeutic adherence, HBP-HL and therapeutic adherence, and therapeutic adherence and HRQoL, were deleted. After optimization, the HRQoL impact factor model (M2) had a better fitting index than the hypothesis model (M1). The optimized model is shown in Fig. 2; it composed of 5 modules and 16 dimensions.

We further evaluated the fitting effect of the path analysis model for HRQoL. When a sample size of 500 was amassed, the model reached the standard, and the fitting indicators met the requirements; this means the model fits well (see Table 4).

**Table 4:** Goodness-of-fit indices of the structural equation model for HRQoL among Kazakh hypertension patients

	χ^**2**^/df	RMSEA	GFI	AGFI	IFI	CFI
M2	2.078	0.046	0.968	0.944	0.949	0.947
Evaluation Criterion	<3.000	<0.080	>0.90	>0.90	>0.90	>0.90

*RMSEA* Root Mean Square Error of Approximation, *GFI* Goodness-of-fit Index, *AGFI* Adjusted Goodness-of-fit Index, *IFI* Incremental fit Index, *CFI* Comparative fit Index

The testing of the modified model showed that all the paths were statistically significant (*P* < 0.05, C.R. > 1.96) and meaningful (Table 5, Path analysis results of HRQoL). Among the paths, HBP-HL had the biggest direct effect on HRQoL (0.350), followed by self-management behavior (0.257), social support (0.190), and self-efficacy (0.183). The model also indicated that self-efficacy could impact HRQoL through self-management behavior.

**Table 5:** Path analysis results of HRQoL among Kazakh hypertension

Regression path	Standardized Estimate*	S.E.	C. R.	***P***
Self-management←Self-efficacy	0.198	0.069	3.371	<0.001
**HRQoL**←HBP-HL	0.350	31.991	2.119	0.034
**HRQoL**← Self-management	0.257	0.856	4.059	<0.001
**HRQoL**← Self-efficacy	0.183	0.650	4.457	<0.001
**HRQoL**←Social Support	0.190	0.187	4.714	<0.001

Standardized Estimate*:A higher score indicted a higher impact for the path

*S. E.* Standard Error in Estimate, *C. R.* Critical Ratio

## Discussion

The results of this study indicate that HRQoL, HBP-HL, and self-management behavior of Kazakh hypertension patients in rural areas are suboptimal, but their self-efficacy and social support status are high. The standardized HRQoL score was 63.30 points, which was at a low level. This result is approximately the same as those in Islamabad, Pakistan (64.56 points) [56]. Among all the dimensions, the physical function was the worst. This result is similar to a study conducted among hypertension patients in Anuradhapura District in North Central Province, Sri Lanka [5]. Tailored interventions should be implemented to improve the HRQoL of Kazakh hypertension patients, especially their physical functions.

Our results showed that the majority of the patients, up to 94% of them, lack HBP-HL. They are unable to read or understand the instructions of the prescription drugs, or can’t communicate effectively with doctors. Research discussed that patients with high HBP-HL have better hypertension control and better HRQoL. The low health literacy causes a 10-year increased risk of cardiovascular disease [57]. Furthermore, Halladay J R et al. demonstrated that health literacy intervention may equally lower SBP in patients with low and higher health literacy [58]. All of these findings indicate the importance of assessing HBP-HL among hypertension patients.

The standardized score of self-management behavior among the Kazakh hypertension patients was at a medium-low level (66.23 points), and it was much lower than the score among hypertension patients in Guangdong, China (86.01points) [59]. Potential reasons for the lower self-management behavior among Kazakh hypertension patients include these factors that all patients in this study living in the remote rural area, and having poor economic situations and education. In addition, since Kazakh is the patients’ main communication language [60], and their Chinese reading and communication ability are relatively low, it creates difficulties in access knowledge and influences their ability to manage the diseases by themselves. Moreover, the patients live in a more dispersed area and often moved to different areas during different seasons, which makes it difficult to assemble the patients and their families, to carry out health educational activities. Mackey L M et al. discussed that low Health Literacy affects the development of self-management skills [61].

Extensive research indicates that health literacy plays an important role in promoting the HRQoL [16, 62, 63]. One study conducted in Tehran, Iran, concluded that health literacy and HRQoL had a significantly positive correlation and suggested that nursing officials and policymakers take measures to promote patients’ HRQoL by improving patients’ health literacy [64]. Previous studies using the SEM in hypertension patients mainly focus on the factors influencing of self-management behavior [59]. Only one article using the SEM explored the relation between health literacy and HRQoL in hypertension patients and found that enhancing the level of health literacy and self-management might improve the HRQoL of patients with hypertension, but the health literacy is universal and lack of pertinence [41].

Our study that introduced hypertension-specific health literacy (HBP-HL) instrument, further explored the direct and indirect effect of factors affecting HRQoL, which might lead to better interventions aimed at ameliorating HRQoL of Kazakh hypertension patients. The indexes of the model reached the adaptation criteria, and the fitting was good. The effects of HBP-HL, social support, self-efficacy, and self-management behavior on HRQoL are positive and statistically significant. HBP-HL has the biggest direct effect on HRQoL. The results of this study are consistent with worldwide results. For example, Tartavoulle study showed that social support had a positive effect on the path of HRQoL [65]. The study by Lee et al. demonstrated that there was a positive effect of self-efficacy on HRQoL [66]. Also, a study among patients with type 2 diabetes showed that self-management, disease-related knowledge, and attitude were the decisive factors that affected the HRQoL [67]. One study conducted by Osborn et al. also found that the health literacy level and health knowledge path of hypertension patients were statistically significant and that health knowledge further affected patients’ self-efficacy and health status [68]^.^ Recent studies strongly suggested that the higher the level of health literacy, the better the HRQoL [69]. These studies do not only support these correlations but further indicate the feasibility of our model. Thus, it seems essential to improve the level of HBP-HL among Kazakh hypertension patients.

There is a positive correlation between therapeutic adherence and HRQoL, however, the path of therapeutic adherence and HRQoL is not statistically significant in SEM. This result is not accordance with the study by Mollaoğlu M, et al. [32], but it is consistent with Hanus J S, et al. [33]. Explanation for the result might be the highly collinear relationship between therapeutic adherence and medication management dimension of self-management behavior. The effect of self-management behavior on HRQoL far outweigh therapeutic adherence.

### Limitations

Some possible limitations of this study should be noted. First, a cross-sectional design limits the causal conclusions that we can draw, and the causal relationship should be confirmed in further prospective studies. Second, Kazakh hypertensive patients were recruited from the same region, which might have limited the generalizability of the study results. Thus, further efforts should be made using multiple strategies to make the sample more comprehensive in future.

## Conclusions

HRQoL, HBP-HL, and self-management behavior of Kazakh hypertension patients in rural areas are poor. The structural equation model of HRQoL works well. HBP-HL has the largest impact on the HRQoL in the model. The results highlight that researchers should give a priority to evaluate patient’s HBP-HL before intervention. Next, tailored interventions are implemented for patients, and ultimately it will contribute to control blood pressure and improve patients’ HRQoL.

## Data Availability

The datasets used and/or analysed during the current study are available from the corresponding author on reasonable request.

## Abbreviations

HRQoL: Health-Related Quality of Life
HBP-HL: High Blood Pressure-Health Literacy
C-HBP-HLS: Chinese-High Blood Pressure-Health Literacy Scale
QLICD-HY: HRQoL Instruments for Chronic Disease-Hypertension
HPSMBRS: Hypertension Patients Self-Management Behavior Rating Scale
TASHP: Therapeutic Adherence Scale for Hypertensive Patients
SSRS: Social Support Rating Scale
RMSEA: Root Mean Square Error of Approximation
GFI: Goodness-of-fit Index
AGFI: Adjusted Goodness-of-fit Index
IFI: Incremental fit Index
CFI: Comparative fit Index
